# The roles of resident, central and effector memory CD4 T‐cells in protective immunity following infection or vaccination

**DOI:** 10.1111/imm.12929

**Published:** 2018-04-17

**Authors:** Joshua I. Gray, Lotus M. Westerhof, Megan K. L. MacLeod

**Affiliations:** ^1^ Centre for Immunobiology Institute of Infection, Immunity and Inflammation University of Glasgow Glasgow UK; ^2^ GLAZgo Discovery Centre Institute of Infection, Immunity and Inflammation University of Glasgow Glasgow UK

**Keywords:** CD4 T cell, cytokine, infection, memory, vaccine

## Abstract

Immunological memory provides rapid protection to pathogens previously encountered through infection or vaccination. CD4 T‐cells play a central role in all adaptive immune responses. Vaccines must, therefore, activate CD4 T‐cells if they are to generate protective immunity. For many diseases, we do not have effective vaccines. These include human immunodeficiency virus (HIV), tuberculosis and malaria, which are responsible for many millions of deaths each year across the globe. CD4 T‐cells play many different roles during the immune response coordinating the actions of many other cells. In order to harness the diverse protective effects of memory CD4 T‐cells, we need to understand how memory CD4 T‐cells are generated and how they protect the host. Here we review recent findings on the location of different subsets of memory CD4 T‐cells that are found in peripheral tissues (tissue resident memory T‐cells) and in the circulation (central and effector memory T‐cells). We discuss the generation of these cells, and the evidence that demonstrates how they provide immune protection in animal and human challenge models.

## Introduction

Vaccines are the most cost‐effective form of healthcare worldwide. Most current vaccines act by generating protective antibodies that inactivate the pathogen or its toxins.^1^ We do not, however, have effective vaccines against pathogens responsible for many millions of deaths each year, such as human immunodeficiency virus (HIV), malaria and tuberculosis. These pathogens present difficult challenges to the immune system either through their complex life cycles and/or via their ability to impair or subvert the host's immune response. Protective immunity to these infections requires a more diverse immune response than antibodies alone. CD4 T‐cells are central to all adaptive immune responses, coordinating pathogen control and clearance by both immune and local stromal cells. Harnessing their diverse functions has the potential to provide novel vaccine strategies that generate effective immunity against even complex infections.

CD4 T‐cells act in concert with innate and other adaptive immune cells to protect the host from pathogens. During primary immune responses, CD4 T‐cells are activated in secondary lymphoid organs where they amplify the anti‐pathogen response by driving B‐cell germinal responses and supporting CD8 T‐cell activation. Activated CD4 T‐cells also migrate from secondary lymphoid organs to inflamed sites where they participate in controlling and/or clearing the pathogen. Following pathogen control or clearance, the vast majority of activated CD4 T‐cells undergo apoptosis with the remainder, usually around 10%, forming a population of long‐lived memory cells. These memory cells retain knowledge about the initial immune response, enabling them to respond more effectively following a secondary infection. This enhanced response forms the basis for the success of vaccines. Understanding the signals that drive the generation of protective memory CD4 T‐cells and the mechanisms by which they act will facilitate the design and development of improved vaccines.

## Cytokine production is key to CD4 T‐cell protective immunity

Naïve CD4 T‐cells can differentiate into a number of distinct functional subsets.^2^, ^3^ This enables them to tailor the immune response depending on the type of pathogen, and to perform multiple functions at different sites during a single infection. Memory CD4 T‐cells retain characteristics of the activated CD4 T‐cells from which they are derived and can, therefore, also be divided based on their functional responses.^4^, ^5^ This cellular memory is thought to be retained by epigenetic changes to the cell's DNA or associated histone proteins that keep genes in an open or closed state depending on their expression during the primary immune response.^3^, ^6^, ^7^ Many genes are thought to be maintained in a poised state in memory T‐cells, enabling rapid re‐expression of effector molecules following T‐cell reactivation by antigen‐presenting cells (APCs). However, epigenetic alterations to the genome are not fixed and memory cells can display plasticity.^8^, ^9^, ^10^, ^11^ The level of this plasticity is likely dependent on the extent of differentiation during the primary response and on the reactivation environment.

Cytokine responses by memory CD4 T‐cells are key to their ability to protect the host from infectious disease. Rapid production of the most appropriate cytokine enables CD4 T‐cells to quickly control the pathogen. For example, interferon (IFN)‐*γ* from CD4 T‐cells protects against viral infections, while interleukin (IL)‐17 aids controls of bacterial and fungal infections, and IL‐4 protects against infection by parasitic worms.^12^, ^13^, ^14^, ^15^, ^16^, ^17^, ^18^, ^19^, ^20^ Most immune protection studies are carried out in animal models where challenge studies are feasible and mechanisms of protection can be identified by loss or gain of function. Human challenge studies are, however, becoming more frequent and have also demonstrated that cytokine‐producing memory CD4 T‐cells correlate with reduced symptoms following pathogen challenge.^21^, ^22^, ^23^

Immune protection by cytokine‐producing CD4 T‐cells correlates with their production of several related cytokines, with cells producing IFN‐*γ*, tumour necrosis factor (TNF)‐*α* and IL‐2 most commonly studied. These multifunctional memory CD4 T‐cells are found following vaccination or infection in animal models and in humans.^24^, ^25^, ^26^, ^27^, ^28^, ^29^ In infection models of *Leishmania major* and *Mycobacterium tuberculosis*, multifunctional CD4 T‐cells provide the most effective immune protection, and in humans they correlate with successful recovery from infection with Japanese encephalitis virus.^25^, ^27^, ^29^

It is currently unclear why multifunctional memory CD4 T‐cells offer enhanced protection in comparison to single cytokine‐producing cells. The enhanced protective functions of these cells could be because they produce higher levels of the individual cytokines on a per cell basis.^25^, ^27^ Alternatively, or in addition, as multifunctional memory T‐cells can simultaneously drive effector responses, for example via IFN‐*γ*, and T‐cell survival and proliferation via IL‐2 production, they offer a sustained and protective response. Currently we have limited understanding of the priming signals that lead to the generation of multifunctional memory cells. This information will be key to the development of more effective vaccines capable of producing protective multifunctional CD4 T‐cells.

## Memory CD4 T‐cells are found throughout the body

**Figure 1:**
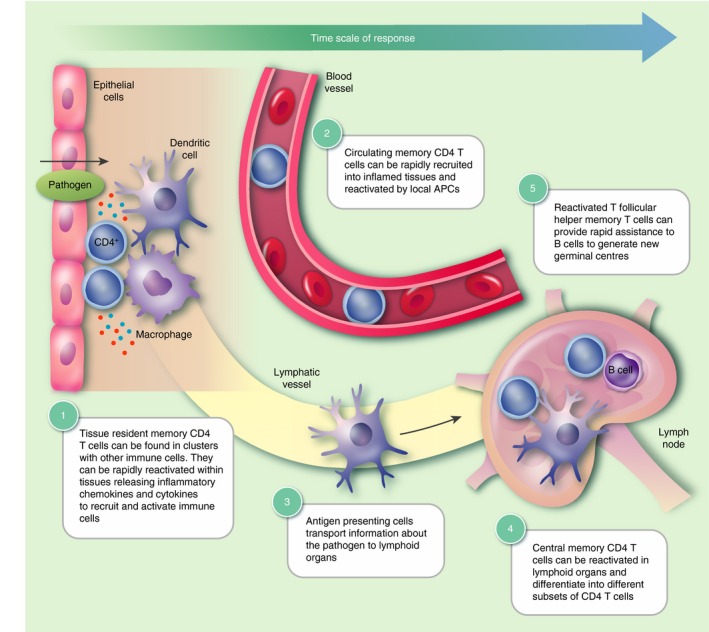
Protective roles of memory CD4 T‐cell subsets. The protective response to previously encountered pathogens is dependent on a number of T‐cell subsets. Upon pathogen encounter, resident memory CD4 T‐cells (Trm) that can be found in clusters with macrophages or dendritic cells respond rapidly by proliferating and releasing inflammatory cytokines and chemokines (1). This stimulates the recruitment of circulating effector memory CD4 T‐cells (Tem) to the inflamed tissue to augment the immune response (2). Local antigen‐presenting cells (APCs) can subsequently transport antigen to lymphoid organs where they activate central memory CD4 T‐cells (Tcm) (3–4). These central memory T‐cells then expand and recirculate to confer systemic protection or further amplify the response at inflamed tissues. Memory follicular helper T‐cells (Tfh) reactivated by either dendritic cells or B‐cells can enter B‐cell zones and induce rapid production of class‐switched antibodies, which are then released into the circulation (5). These different memory T‐cell subsets work in concert to provide long‐lasting protection upon re‐exposure to the same pathogen.

The consequences of rapid cytokine production by memory CD4 T‐cells depend on the location of the cell. Naïve T‐cells patrol through lymphoid organs as they have no prior knowledge about where in the body the pathogen they recognize may cause an infection. Memory T‐cells have learnt this information during the primary response, and some memory CD4 T‐cells, tissue resident memory (Trm) cells, continue to reside at the infection site. Other memory T‐cells recirculate through the body. These memory cells can be split into central memory cells (Tcm) and effector memory cells (Tem). Tcm are largely restricted to lymphoid organs and the blood. In contrast, Tem are present in the blood and have the ability to traffic through peripheral organs. All types of memory CD4 T‐cells are important. This is because they can provide protection in distinct ways, with recirculating cells acting as reinforcements should resident cells fail to contain the infection (Fig. 1.

### Trm T‐cells

The anti‐pathogen responses coordinated by naïve T‐cells are delayed until information about the infection is transferred from the infection site to the draining lymph node. Trm cells can provide much more immediate protection, and various studies have demonstrated that they provide the most effective immune protection to the host.^14^, ^15^, ^17^, ^30^

Tissue resident memory cells are identified as CD69+ cells that remain within peripheral tissues following pathogen clearance.^30^, ^31^, ^32^ CD69 is thought to act as a retention signal as it inhibits the surface expression of the sphingosine‐1‐phosphate (S1P) receptor 1. S1P is a signalling phospholipid that regulates the migration of immune cells out of lymph nodes into efferent lymphatics, and also guides cells out of tissues towards draining lymph nodes.^33^, ^34^, ^35^ Expression of the integrin CD103 by CD8 Trm cells also contributes to their retention, tethering them to skin or mucosal epithelial cells; it is, however, more rare for CD4 Trm cells to express this molecule.^36^

There are clear differences between CD4 and CD8 Trm cells first revealed by Gebhardt *et al*. in mice infected with herpes simplex virus (HSV).^14^ While CD8 Trm cells become dendritic‐like with limited motility in the epithelium of the skin, CD4 Trm cells are mainly found in the dermis and display a more motile behaviour.^14^ In humans, Trm cells have been found in multiple organs, with elegant studies from Farber and colleagues tracking memory T‐cells in individuals of various ages. Human and mouse Trm cells share many characteristics, including CD69 expression and reduced expression of S1P receptor 1.^37^, ^38^ Similarly, CD4 Trm cells in skin are, like their mouse equivalent, mainly found in the dermis.^39^ Transcriptional analyses comparing circulating cells and Trm cells from the same donor indicate that CD69+ and CD69− CD4 T‐cells express overlapping sets of T‐cell receptor genes.^37^ These data indicate that environment, rather than epitope specificity, is the key driver of Trm cell development.

In mice, memory T‐cell residency is often identified by their failure to bind fluorescently labelled antibody injected intravenously shortly before the animal is killed. This demonstrates that these cells are not directly in contact with the vasculature, at least at the time of analysis. Three additional key approaches are used in mice to investigate whether Trm cells are truly resident: *in vivo* treatment with the S1P functional antagonist, FTY720; parabiosis in which the circulatory systems of two animals are surgically connected; and fate mapping using photoconvertible cells. Treatment with FTY720 restricts the migration of circulating cells, so a stable population of Trm cells in treated mice demonstrates that these cells are neither replenished by, nor lost to, circulating populations.^15^, ^17^, ^40^ However, FTY720 may also inhibit egress of cells from peripheral tissues to draining lymph nodes and/or reduce cell survival.^35^, ^41^, ^42^, ^43^ Despite these potential caveats, results from FTY720‐treated animals reflect those from more elegant parabiosis experiments that demonstrate that Trm cells are a distinct population neither leaving the tissue nor being replenished by circulating cells.^17^, ^30^

In contrast, data from Collins *et al*. suggest that CD4 Trm cells are a more dynamic population.^44^ CD4 Trm cells in mice that express the photoconvertible molecule, Kaede, were found to migrate from the skin of HSV‐infected mice to draining lymph nodes. These cells lost expression of CD69 following migration out of the skin. The migrating cells represented about half of the Trm cell population photoconverted at the start of the study and then examined 3 days later. These findings question the reliability of CD69 as a marker of persistently resident memory T‐cells within tissues. They suggest that at least two distinct populations may be present – a more static, true Trm population and a second more dynamic population that alter CD69 expression depending on location. In this study, the specificity of the cells was not determined. These two populations may, therefore, reflect resident T‐cells that respond to local antigen and remain in the tissue versus cells that are recruited but are not maintained because they are not re‐activated by antigen. Future studies will need to address the functional properties of these two populations and compare the behaviours of T‐cells exposed to local antigen versus those recruited by inflammation alone.

To develop vaccines that generate protective CD4 Trm cells, it is imperative that we understand the signals that drive the development and maintenance of these cells. During immune responses, activated T‐cells migrate into inflamed tissues under the guidance of chemokines, integrins and adhesion molecules.^45^ T‐cells that encounter their antigen at the infection site are likely to receive tissue‐specific cues that influence their function and memory potential. The presentation of specific antigen at the infected tissue is required for the formation of CD4 Trm cells, but may not be necessary for CD8 Trm cells.^31^, ^46^, ^47^, ^48^ These data indicate that vaccines aimed at generating protective Trm CD4 T‐cells must drive antigen presentation within the tissue targeted by the pathogen.

Whether persistent antigen presentation following pathogen control is required to maintain CD4 Trm cells is still unclear. CD4 Trm cells in the skin and mucosa are located in clusters of cells with macrophages and/or dendritic cells that express chemokines that maintain the cells at the site. Macrophage‐derived CCL5 maintains CD4 T‐cells in the vagina following HSV‐2 infection.^17^ IFN‐*γ* produced by CD4 Trm cells, potentially in response to low levels of persistent antigen, in turn maintains macrophage CCL5 expression. Similarly, CCL5 produced by CD8 T‐cells and macrophages in the skin is responsible for maintaining CD4 T‐cells in perifollicular clusters.^44^ In this case, however, antigen presentation was not required for memory T‐cells to be incorporated into these immune cell clusters.

Clusters of Trm and APCs may represent nascent versions of ectopic lymphoid structures (ELS), which range from organized clusters of immune cells to lymph node‐like structures with designated T‐ and B‐cell zones. ELS are often found in sites of chronic inflammation, and have been associated with the generation of autoreactive antibodies.^49^, ^50^ Whether Trm immune cell clusters represent an early stage in ELS development is unclear. They do provide an excellent location for the rapid reactivation of CD4 Trm cells by local APCs. For example, influenza virus‐specific Trm cells, which can be found in clusters with B‐cells in the infected lung, provide effective and rapid immune protection at least in part by providing rapid assistance to B‐cells to make neutralizing antibodies.^18^

In addition, CD4 Trm cells can enhance the actions of cells that are recruited into the infected tissue. Influenza‐specific memory CD4 T‐cells in the lung drive the production of chemokines that attract innate immune cells that rapidly control viral spread.^51^ Similarly, IFN‐*γ* production by *L. major*‐specific CD4 Trm cells drives the recruitment of inflammatory monocytes to the infection site.^15^ These recruited monocytes produce nitric oxide and reactive oxygen species that are toxic to the parasite.

### Recirculating memory T‐cells

Circulating antigen‐specific CD4 memory T‐cells can participate in protective immune responses either acting as reinforcements to Trm cells^32^ or protecting tissues not challenged in the initial infection or vaccination.^14^, ^52^ Immune protection by circulating memory CD4 T‐cells is delayed compared with Trm‐mediated protection as they must be first recruited to the site of infection and reactivated by local APCs. Immune protection by circulating cells can require collaboration with other components of the memory immune response. Iijima *et al*. found that while recirculating HSV‐2‐specific memory CD4 T‐cells cannot prevent viral replication at the challenge site, they could prevent virus entry into the dorsal route ganglia.^52^ This protection required the entry of virus‐specific antibody into the nervous system, which was dependent on increased vascularization mediated by IFN‐*γ* derived from reactivated memory CD4 T‐cells. Similarly, in mice vaccinated with the yellow fever vaccine, transfer of CD4 T‐cells and immune serum provide the most optimal immune protection.^20^

The effector response of recruited antigen‐specific memory CD4 T‐cells is likely to be influenced by the level of MHC II and co‐stimulatory molecules displayed by the local APC that reactivates the memory cell.^53^, ^54^, ^55^ Furthermore, this response is likely to be distinct to that from the same cell reactivated in the very different environment of a lymphoid organ. It is key, therefore, to consider not just the type of memory CD4 T‐cell that a vaccine should aim to generate but where it is likely to be reactivated, which APCs are involved in this, and the downstream consequences of these interactions.

### Memory CD4 T‐cells in secondary lymphoid organs

Central memory cells are most likely to be reactivated in secondary lymphoid organs as they lack the chemokine receptors and adhesion molecules necessary to enter peripheral tissues.^45^ Instead they, like naïve T‐cells, express high levels of CD62L, which enables entry into lymph nodes from the blood via high endothelial venules and CCR7, the chemokine receptor that is also involved in trafficking to and within lymphoid organs.^45^, ^56^

While Tcm cells may not rapidly produce protective cytokines, they proliferate upon reactivation, quickly increasing the number of antigen‐specific CD4 T‐cells. These cells can then either remain in the lymphoid organ to help B‐cells or migrate to the site of infection to help tackle the infection directly. As Tcm cells are uncommitted to any particular effector cytokine production, they can differentiate under the influence of the cytokine milieu triggered by the challenge infection.^57^ Vaccines that drive the generation of Tcm cells may provide less immediate protection than those designed to induce Trm cells; however, they offer an adaptable pool of memory CD4 T‐cells that can protect via multiple pathways.

In primary immune responses, activated CD4 T‐cells drive B‐cell germinal centre reactions leading to the production of high‐affinity class‐switched antibody. The cells that coordinate this response are classed as T follicular helper (Tfh) cells that are initially formed in the T‐cell zone of the lymphoid organ before moving to the developing germinal centre.^7^ Depending on the type of infection, Tfh cells can produce T helper type 1 or 2 cytokines to drive appropriate antibody class switching.^7^

Whether Tfh cells can differentiate into specialized memory cells has been an area of recent debate.^7^ Within the pool of Tcm cells, a proportion of cells express higher levels of CXCR5, the chemokine receptor that allows cells to move towards or into B‐cell follicles.^7^ Tfh cells can remain for many months in the original draining lymph node where persistent antigen is likely to maintain them in an active state. These cells rapidly expand upon re‐challenge and may represent a population of lymph node resident memory cells as they can express CD69.^58^, ^59^, ^60^ However, this reliance on antigen suggests that these lymph node resident Tfh cells may not represent ‘true resting memory cells’. Moreover, Pepper *et al*. found that the number of antigen‐specific memory cells that expressed Tfh markers declined over time, suggesting that Tfh cells fail to differentiate into long‐lived memory cells.^57^ However, we and others have shown that both mouse and human CXCR5+ memory CD4 T‐cells provide rapid assistance to B‐cells upon reactivation.^61^, ^62^, ^63^, ^64^, ^65^, ^66^, ^67^, ^68^, ^69^ Importantly, Alexander *et al*. demonstrated that DNA vaccination generated memory CD4 T‐cells that, via their rapid assistance to B‐cells, protected mice from influenza virus infection.^70^ This rapid assistance to generate high‐affinity class‐switched antibody is particularly relevant to infections such as influenza virus where regions of the virus targeted by antibody alter much more rapidly than epitopes recognized by CD4 T‐cells.^71^

CXCR5+ memory cells with an increased ability to help B‐cells are commonly referred to as Tfh memory cells,^7^ even though they may be found in circulation rather than contained within a B‐cell follicle. How they are distinct from the general CD4 Tcm pool is a complicated question. Indeed, Tfh memory cells do express lower levels of CXCR5 than Tfh cells present during the primary immune response^62^, ^69^ and, as Pepper *et al*. describe, are difficult to distinguish from Tcm cells.^57^ Reactivation of CXCR5+ memory cells can occur in the absence of B‐cells,^63^ but reactivation by B‐cells consolidates the T‐cell's expression of Bcl6, the transcription factor associated with Tfh cell function.^72^ These data suggest that if there are Tfh memory cells, their function upon reactivation is likely dependent on the context in which they are reactivated.

A further key question that remains to be addressed is the consequences of reactivation of memory CD4 T‐cells by B‐cells within either ELS or immune cell clusters at the infection site itself. In lymphoid organs, secondary germinal centres are thought to be formed by reactivated IgM+ memory B‐cells, providing a blank canvas for antibody class switching relevant to the pathogen.^73^ In influenza virus‐infected mice, CD4 T‐cells and B‐cells can be found in clusters within the lung, and many virus‐specific B‐cells in the lung are class switched.^74^, ^75^ This suggests that germinal centres formed in ELS in peripheral tissues may follow different rules to those in lymphoid organs following re‐infection. Careful studies that dissect the contribution of the reactivation of B‐cells in the tissue versus those in lymph nodes to immune protection are needed to establish their relative importance in protecting the host from reinfection.

## Human vaccines: recent progress and continuing challenges

Dissecting the relative contributions of different populations of memory T‐cells to immune protection in mice provides mechanistic understanding of immunological memory. Animal vaccine studies often do not, however, easily translate into protective vaccines for humans. Human challenge studies, therefore, play important roles in evaluating vaccines at early stages of development. They often cut the costs of large‐scale field vaccine trials and reduce tests of non‐effective vaccines on large numbers of individuals.^76^ This is especially the case in diseases, including malaria and tuberculosis, in which our understanding of the correlates and mechanisms of immune protection are limited.

Human challenge studies in malaria have demonstrated the relative safety and efficacy of sporozoite vaccines. Experimental sporozoite vaccines have either been delivered as irradiated parasites that can infect host cells but fail to differentiate and cause disease, or as live parasites.^21^, ^77^, ^78^ In this instance, anti‐malarial drugs must be given to prevent active disease. Protection in these studies is associated with high levels of multifunctional, antigen‐specific memory CD4 T‐cells in peripheral blood, although mouse and non‐human primate studies point to key roles for liver resident memory CD8 T‐cells in preventing parasite growth.^78^, ^79^ The logistical challenges of sporozoite vaccines are significant; current field trials are likely to reveal whether or not these approaches are feasible.^80^

The most advanced vaccine for malaria is GlaxoSmithKline's RTS,S vaccine, which contains the circumsporozoite protein from the pre‐erythrocyte stage of the parasite.^81^, ^82^ Antibodies and CD4 T‐cells specific to the circumsporozoite protein are correlates of immune protection following RTS,S immunization, which has an efficacy of about 30%.^22^, ^81^, ^83^ While a positive advance, this relatively low efficacy and the short duration of protection means that more robust vaccines are still required.^84^ A key contrast between sporozoite vaccines and the recombinant protein RTS,S vaccine is the need for adjuvants to boost the immune response to the subunit vaccine. RTS,S contains a combined adjuvant with a saponin and toll‐like receptor agonist.^82^ This adjuvant combination is one of only a handful approved for use in humans, with few able to induce the size and correctly tailored immune response driven by either natural infection or vaccination with an attenuated or inactivated pathogen.^82^ The live vaccines are, however, more likely to lead to adverse effects or negative side‐effects.^82^, ^85^ Improved understanding of the mechanisms of action of current and experimental adjuvants is likely to lead to further improvements allowing us to strike the right balance between immunogenicity and vaccine safety.

Other human challenge models include the use of BCG as a surrogate challenge in tuberculosis vaccine trials. While the BCG vaccine protects against disseminated tuberculosis, especially in young children who are most at risk, its ability to protect from pulmonary disease varies across the world.^86^ Current studies in mice, non‐human primates and humans are focussed on increasing the immune protection offered by BCG, for example by using prime‐boost strategies.^86^, ^87^ BCG effectiveness can also be achieved by altering the vaccination route, with mucosal and intravenous routes offering enhanced protection compared with more traditional subcutaneous or intradermal injection in a non‐human primate model.^88^ Potentially these injection routes provide enhanced protection as they are better at driving the development of CD4 Trm cells in the lung.

Human immunodeficiency virus presents bigger hurdles still as immune protection does not develop in the vast majority of infected individuals and human challenge studies are not possible. The RV144 HIV vaccine trial has offered a number of key insights into immunity to HIV. The vaccine demonstrated an efficacy of 31·2%.^89^ Protection correlated with high levels of anti‐viral IgG, dependent on CD4 T‐cell responses, while vaccine‐driven IgA was associated with an increased risk of infection.^90^, ^91^, ^92^ Immune protection to this highly diverse virus is thought to require broadly neutralizing antibodies (bnAbs) that recognize more conserved regions of the virus.^93^ The development of bnAbs likely requires repeated exposure to the antigen and continued input from Tfh cells.^93^ Successful HIV vaccines will probably, therefore, require the induction of effective Tfh memory cells that can coordinate the development of protective bnAbs.

## Future perspectives

We have appreciated the concept of protective immunological memory for thousands of years and have been manipulating it via vaccination since the 10th century.^85^ The majority of successful vaccines act by generating neutralizing class‐switched antibodies produced by long‐lived plasma cells.^1^ These vaccines must, therefore, drive activation of helper CD4 T‐cells that act, at the very least, during the primary response to protect the host. We are now beginning to understand the potential myriad roles memory CD4 T‐cells can themselves play in protection from infectious diseases. The recent studies discussed here have revealed the complexity of the memory CD4 T‐cell pool. We now need to understand how vaccine formation and delivery can be altered to bias the development of protective memory CD4 T‐cells.

As our fundamental understanding of memory CD4 T‐cells improves, we must put this into a real‐world context. Studies in wild or pet‐shop mice have highlighted the major differences between memory compartments and responses to infections in these animals versus their laboratory equivalent.^94^, ^95^ The microbiome plays a major role in these differences, but the exposure to varied and multiple pathogens throughout life is also likely to affect the generation and function of immune memory cells.^94^, ^95^ Human challenge studies offer a valuable extra arm to vaccine studies, but most are carried out on healthy western volunteers. Evidence from the recently introduced rotavirus vaccine, and also polio and cholera vaccine studies, demonstrates reduced vaccine‐effectiveness in major at‐risk populations in low–middle‐income countries.^96^, ^97^, ^98^ It is clear, therefore, that protective immunity must be examined and evaluated in multiple experimental settings that can each provide valuable information on both mechanisms of immune protection and real‐world effectiveness.

## Disclosures

The authors declare that they have no competing interests.

## References

[1] Plotkin SA . Correlates of protection induced by vaccination. Clin Vaccine Immunol 2010; 17:1055–65. 20463105 10.1128/CVI.00131-10 PMC2897268

[2] O'Shea JJ , Paul WE . Mechanisms underlying lineage commitment and plasticity of helper CD4+ T cells. Science 2010; 327:1098–102. 20185720 10.1126/science.1178334 PMC2997673

[3] Wang C , Collins M , Kuchroo VK . Effector T cell differentiation: are master regulators of effector T cells still the masters? Curr Opin Immunol 2015; 37:6–10. 26319196 10.1016/j.coi.2015.08.001

[4] Jaigirdar SA , MacLeod MK . Development and function of protective and pathologic memory CD4 T cells. Front Immunol 2015; 6:456. 26441961 10.3389/fimmu.2015.00456 PMC4561815

[5] Mueller SN , Gebhardt T , Carbone FR , Heath WR . Memory T cell subsets, migration patterns, and tissue residence. Annu Rev Immunol 2013; 31:137–61. 23215646 10.1146/annurev-immunol-032712-095954

[6] Bevington SL , Cauchy P , Withers DR , Lane PJ , Cockerill PN . T cell receptor and cytokine signaling can function at different stages to establish and maintain transcriptional memory and enable T helper cell differentiation. Front Immunol 2017; 8:204. 28316598 10.3389/fimmu.2017.00204 PMC5334638

[7] Hale JS , Ahmed R . Memory T follicular helper CD4 T cells. Front Immunol 2015; 6:16. 25699040 10.3389/fimmu.2015.00016 PMC4313784

[8] Ahmadzadeh M , Farber DL . Functional plasticity of an antigen‐specific memory CD4 T cell population. Proc Natl Acad Sci USA 2002; 99:11 802–7. 10.1073/pnas.192263099 PMC129349 12192093

[9] Krawczyk CM , Shen H , Pearce EJ . Functional plasticity in memory T helper cell responses. J Immunol 2007; 178:4080–8. 17371962 10.4049/jimmunol.178.7.4080

[10] Lee YK , Turner H , Maynard CL , Oliver JR , Chen D , Elson CO , *et al* Late developmental plasticity in the T helper 17 lineage. Immunity 2009; 30:92–107. 19119024 10.1016/j.immuni.2008.11.005 PMC3607320

[11] Wei G , Wei L , Zhu J , Zang C , Hu‐Li J , Yao Z , *et al* Global mapping of H3K4me3 and H3K27me3 reveals specificity and plasticity in lineage fate determination of differentiating CD4+ T cells. Immunity 2009; 30:155–67. 19144320 10.1016/j.immuni.2008.12.009 PMC2722509

[12] Anthony RM , Urban JF Jr , Alem F , Hamed HA , Rozo CT , Boucher JL , *et al* Memory T(H)2 cells induce alternatively activated macrophages to mediate protection against nematode parasites. Nat Med 2006; 12:955–60. 16892038 10.1038/nm1451 PMC1955764

[13] Brown DM , Lee S , Garcia‐Hernandez Mde L , Swain SL . Multifunctional CD4 cells expressing gamma interferon and perforin mediate protection against lethal influenza virus infection. J Virol 2012; 86:6792–803. 22491469 10.1128/JVI.07172-11 PMC3393557

[14] Gebhardt T , Whitney PG , Zaid A , Mackay LK , Brooks AG , Heath WR , *et al* Different patterns of peripheral migration by memory CD4+ and CD8+ T cells. Nature 2011; 477:216–9. 21841802 10.1038/nature10339

[15] Glennie ND , Volk SW , Scott P . Skin‐resident CD4+ T cells protect against Leishmania major by recruiting and activating inflammatory monocytes. PLoS Pathog 2017; 13:e1006349. 28419151 10.1371/journal.ppat.1006349 PMC5409171

[16] Hernandez‐Santos N , Huppler AR , Peterson AC , Khader SA , McKenna KC , Gaffen SL . Th17 cells confer long‐term adaptive immunity to oral mucosal Candida albicans infections. Mucosal Immunol 2013; 6:900–10. 23250275 10.1038/mi.2012.128 PMC3608691

[17] Iijima N , Iwasaki A . T cell memory. A local macrophage chemokine network sustains protective tissue‐resident memory CD4 T cells. Science 2014; 346:93–8. 25170048 10.1126/science.1257530 PMC4254703

[18] McKinstry KK , Strutt TM , Kuang Y , Brown DM , Sell S , Dutton RW , *et al* Memory CD4+ T cells protect against influenza through multiple synergizing mechanisms. J Clin Invest 2012; 122:2847–56. 22820287 10.1172/JCI63689 PMC3408751

[19] Wang Y , Jiang B , Guo Y , Li W , Tian Y , Sonnenberg GF , *et al* Cross‐protective mucosal immunity mediated by memory Th17 cells against Streptococcus pneumoniae lung infection. Mucosal Immunol 2017; 10:250–9. 27118490 10.1038/mi.2016.41 PMC5083242

[20] Watson AM , Lam LK , Klimstra WB , Ryman KD . The 17D‐204 vaccine strain‐induced protection against virulent yellow fever virus is mediated by humoral immunity and CD4+ but not CD8+ T cells. PLoS Pathog 2016; 12:e1005786. 27463517 10.1371/journal.ppat.1005786 PMC4962991

[21] Mordmuller B , Surat G , Lagler H , Chakravarty S , Ishizuka AS , Lalremruata A , *et al* Sterile protection against human malaria by chemoattenuated PfSPZ vaccine. Nature 2017; 542:445–9. 28199305 10.1038/nature21060 PMC10906480

[22] White MT , Bejon P , Olotu A , Griffin JT , Riley EM , Kester KE , *et al* The relationship between RTS, S vaccine‐induced antibodies, CD4(+) T cell responses and protection against Plasmodium falciparum infection. PLoS ONE 2013; 8:e61395. 23613845 10.1371/journal.pone.0061395 PMC3628884

[23] Wilkinson TM , Li CK , Chui CS , Huang AK , Perkins M , Liebner JC , *et al* Preexisting influenza‐specific CD4+ T cells correlate with disease protection against influenza challenge in humans. Nat Med 2012; 18:274–80. 22286307 10.1038/nm.2612

[24] Bonduelle O , Carrat F , Luyt CE , Leport C , Mosnier A , Benhabiles N , *et al* Characterization of pandemic influenza immune memory signature after vaccination or infection. J Clin Invest 2014; 124:3129–36. 24911149 10.1172/JCI74565 PMC4071387

[25] Darrah PA , Patel DT , De Luca PM , Lindsay RW , Davey DF , Flynn BJ , *et al* Multifunctional TH1 cells define a correlate of vaccine‐mediated protection against Leishmania major. Nat Med 2007; 13:843–50. 17558415 10.1038/nm1592

[26] Harari A , Vallelian F , Meylan PR , Pantaleo G . Functional heterogeneity of memory CD4 T cell responses in different conditions of antigen exposure and persistence. J Immunol 2005; 174:1037–45. 15634928 10.4049/jimmunol.174.2.1037

[27] Lindenstrom T , Agger EM , Korsholm KS , Darrah PA , Aagaard C , Seder RA , *et al* Tuberculosis subunit vaccination provides long‐term protective immunity characterized by multifunctional CD4 memory T cells. J Immunol 2009; 182:8047–55. 19494330 10.4049/jimmunol.0801592

[28] Nelson RW , McLachlan JB , Kurtz JR , Jenkins MK . CD4+ T cell persistence and function after infection are maintained by low‐level peptide:MHC class II presentation. J Immunol 2013; 190:2828–34. 23382562 10.4049/jimmunol.1202183 PMC3594488

[29] Turtle L , Bali T , Buxton G , Chib S , Chan S , Soni M , *et al* Human T cell responses to Japanese encephalitis virus in health and disease. J Exp Med 2016; 213:1331–52. 27242166 10.1084/jem.20151517 PMC4925015

[30] Teijaro JR , Turner D , Pham Q , Wherry EJ , Lefrancois L , Farber DL . Cutting edge: tissue‐retentive lung memory CD4 T cells mediate optimal protection to respiratory virus infection. J Immunol 2011; 187:5510–4. 22058417 10.4049/jimmunol.1102243 PMC3221837

[31] Thom JT , Weber TC , Walton SM , Torti N , Oxenius A . The salivary gland acts as a sink for tissue‐resident memory CD8(+) T cells, facilitating protection from local cytomegalovirus infection. Cell Rep 2015; 13:1125–36. 26526997 10.1016/j.celrep.2015.09.082

[32] Zens KD , Chen JK , Guyer RS , Wu FL , Cvetkovski F , Miron M , *et al* Reduced generation of lung tissue‐resident memory T cells during infancy. J Exp Med 2017; 214:2915–32. 28855242 10.1084/jem.20170521 PMC5626403

[33] Cyster JG , Schwab SR . Sphingosine‐1‐phosphate and lymphocyte egress from lymphoid organs. Annu Rev Immunol 2012; 30:69–94. 22149932 10.1146/annurev-immunol-020711-075011

[34] Czeloth N , Bernhardt G , Hofmann F , Genth H , Forster R . Sphingosine‐1‐phosphate mediates migration of mature dendritic cells. J Immunol 2005; 175:2960–7. 16116182 10.4049/jimmunol.175.5.2960

[35] Matloubian M , Lo CG , Cinamon G , Lesneski MJ , Xu Y , Brinkmann V , *et al* Lymphocyte egress from thymus and peripheral lymphoid organs is dependent on S1P receptor 1. Nature 2004; 427:355–60. 14737169 10.1038/nature02284

[36] Mueller SN , Mackay LK . Tissue‐resident memory T cells: local specialists in immune defence. Nat Rev Immunol 2016; 16:79–89. 26688350 10.1038/nri.2015.3

[37] Kumar BV , Ma W , Miron M , Granot T , Guyer RS , Carpenter DJ , *et al* Human tissue‐resident memory T cells are defined by core transcriptional and functional signatures in lymphoid and mucosal sites. Cell Rep 2017; 20:2921–34. 28930685 10.1016/j.celrep.2017.08.078 PMC5646692

[38] Thome JJ , Yudanin N , Ohmura Y , Kubota M , Grinshpun B , Sathaliyawala T , *et al* Spatial map of human T cell compartmentalization and maintenance over decades of life. Cell 2014; 159:814–28. 25417158 10.1016/j.cell.2014.10.026 PMC4243051

[39] Watanabe R , Gehad A , Yang C , Scott LL , Teague JE , Schlapbach C , *et al* Human skin is protected by four functionally and phenotypically discrete populations of resident and recirculating memory T cells. Sci Transl Med 2015; 7:279ra39. 10.1126/scitranslmed.3010302 PMC4425193 25787765

[40] Turner DL , Bickham KL , Thome JJ , Kim CY , D'Ovidio F , Wherry EJ , *et al* Lung niches for the generation and maintenance of tissue‐resident memory T cells. Mucosal Immunol 2014; 7:501–10. 24064670 10.1038/mi.2013.67 PMC3965651

[41] Jaigirdar SA , Benson RA , Elmesmari A , Kurowska‐Stolarska MS , McInnes IB , Garside P , *et al* Sphingosine‐1‐phosphate promotes the persistence of activated CD4 T Cells in inflamed sites. Front Immunol 2017; 8:1627. 29225602 10.3389/fimmu.2017.01627 PMC5705559

[42] Mendoza A , Fang V , Chen C , Serasinghe M , Verma A , Muller J , *et al* Lymphatic endothelial S1P promotes mitochondrial function and survival in naive T cells. Nature 2017; 546:158–61. 28538737 10.1038/nature22352 PMC5683179

[43] Rathinasamy A , Czeloth N , Pabst O , Forster R , Bernhardt G . The origin and maturity of dendritic cells determine the pattern of sphingosine 1‐phosphate receptors expressed and required for efficient migration. J Immunol 2010; 185:4072–81. 20826749 10.4049/jimmunol.1000568

[44] Collins N , Jiang X , Zaid A , Macleod BL , Li J , Park CO , *et al* Skin CD4(+) memory T cells exhibit combined cluster‐mediated retention and equilibration with the circulation. Nat Commun 2016; 7:11 514. 10.1038/ncomms11514 PMC4866325 27160938

[45] Masopust D , Schenkel JM . The integration of T cell migration, differentiation and function. Nat Rev Immunol 2013; 13:309–20. 23598650 10.1038/nri3442

[46] Mackay LK , Stock AT , Ma JZ , Jones CM , Kent SJ , Mueller SN , *et al* Long‐lived epithelial immunity by tissue‐resident memory T (TRM) cells in the absence of persisting local antigen presentation. Proc Natl Acad Sci USA 2012; 109:7037–42. 22509047 10.1073/pnas.1202288109 PMC3344960

[47] Shin H , Iwasaki A . A vaccine strategy that protects against genital herpes by establishing local memory T cells. Nature 2012; 491:463–7. 23075848 10.1038/nature11522 PMC3499630

[48] Takamura S , Yagi H , Hakata Y , Motozono C , McMaster SR , Masumoto T , *et al* Specific niches for lung‐resident memory CD8+ T cells at the site of tissue regeneration enable CD69‐independent maintenance. J Exp Med 2016; 213:3057–73. 27815325 10.1084/jem.20160938 PMC5154946

[49] Corsiero E , Nerviani A , Bombardieri M , Pitzalis C . Ectopic lymphoid structures: powerhouse of autoimmunity. Front Immunol 2016; 7:430. 27799933 10.3389/fimmu.2016.00430 PMC5066320

[50] Jones GW , Jones SA . Ectopic lymphoid follicles: inducible centres for generating antigen‐specific immune responses within tissues. Immunology 2016; 147:141–51. 26551738 10.1111/imm.12554 PMC4717241

[51] Strutt TM , McKinstry KK , Dibble JP , Winchell C , Kuang Y , Curtis JD , *et al* Memory CD4+ T cells induce innate responses independently of pathogen. Nat Med 2010; 16:558–64. 20436484 10.1038/nm.2142 PMC2927232

[52] Iijima N , Iwasaki A . Access of protective antiviral antibody to neuronal tissues requires CD4 T‐cell help. Nature 2016; 533:552–6. 27225131 10.1038/nature17979 PMC4883597

[53] David A , Crawford F , Garside P , Kappler JW , Marrack P , MacLeod M . Tolerance induction in memory CD4 T cells requires two rounds of antigen‐specific activation. Proc Natl Acad Sci USA 2014; 111:7735–40. 24821788 10.1073/pnas.1406218111 PMC4040609

[54] Mahajan S , Cervera A , MacLeod M , Fillatreau S , Perona‐Wright G , Meek S , *et al* The role of ICOS in the development of CD4 T cell help and the reactivation of memory T cells. Eur J Immunol 2007; 37:1796–808. 17549732 10.1002/eji.200636661 PMC2699381

[55] Riccomi A , Palma C . B Cells and programmed death‐ligand 2 signaling are required for maximal interferon‐gamma recall response by splenic CD4(+) memory T Cells of mice vaccinated with mycobacterium tuberculosis Ag85B. PLoS ONE 2015; 10:e0137783. 26379242 10.1371/journal.pone.0137783 PMC4574766

[56] Sallusto F , Lenig D , Forster R , Lipp M , Lanzavecchia A . Two subsets of memory T lymphocytes with distinct homing potentials and effector functions. Nature 1999; 401:708–12. 10537110 10.1038/44385

[57] Pepper M , Pagan AJ , Igyarto BZ , Taylor JJ , Jenkins MK . Opposing signals from the Bcl6 transcription factor and the interleukin‐2 receptor generate T helper 1 central and effector memory cells. Immunity 2011; 35:583–95. 22018468 10.1016/j.immuni.2011.09.009 PMC3208313

[58] Fazilleau N , Eisenbraun MD , Malherbe L , Ebright JN , Pogue‐Caley RR , McHeyzer‐Williams LJ , *et al* Lymphoid reservoirs of antigen‐specific memory T helper cells. Nat Immunol 2007; 8:753–61. 17529982 10.1038/ni1472

[59] Marriott CL , Dutton EE , Tomura M , Withers DR . Retention of Ag‐specific memory CD4(+) T cells in the draining lymph node indicates lymphoid tissue resident memory populations. Eur J Immunol 2017; 47:860–71. 28295233 10.1002/eji.201646681 PMC5435927

[60] Ugur M , Schulz O , Menon MB , Krueger A , Pabst O . Resident CD4+ T cells accumulate in lymphoid organs after prolonged antigen exposure. Nat Commun 2014; 5:4821. 25189091 10.1038/ncomms5821

[61] Chevalier N , Jarrossay D , Ho E , Avery DT , Ma CS , Yu D , *et al* CXCR5 expressing human central memory CD4 T cells and their relevance for humoral immune responses. J Immunol 2011; 186:5556–68. 21471443 10.4049/jimmunol.1002828

[62] Choi YS , Yang JA , Yusuf I , Johnston RJ , Greenbaum J , Peters B , *et al* Bcl6 expressing follicular helper CD4 T cells are fate committed early and have the capacity to form memory. J Immunol 2013; 190:4014–26. 23487426 10.4049/jimmunol.1202963 PMC3626566

[63] Hale JS , Youngblood B , Latner DR , Mohammed AU , Ye L , Akondy RS , *et al* Distinct memory CD4+ T cells with commitment to T follicular helper‐ and T helper 1‐cell lineages are generated after acute viral infection. Immunity 2013; 38:805–17. 23583644 10.1016/j.immuni.2013.02.020 PMC3741679

[64] Liu X , Yan X , Zhong B , Nurieva RI , Wang A , Wang X , *et al* Bcl6 expression specifies the T follicular helper cell program in vivo. J Exp Med 2012; 209:S1–24. 10.1084/jem.20120219 PMC3457730 22987803

[65] Locci M , Havenar‐Daughton C , Landais E , Wu J , Kroenke MA , Arlehamn CL , *et al* Human circulating PD‐1+CXCR3‐CXCR5+ memory Tfh cells are highly functional and correlate with broadly neutralizing HIV antibody responses. Immunity 2013; 39:758–69. 24035365 10.1016/j.immuni.2013.08.031 PMC3996844

[66] MacLeod MK , David A , McKee AS , Crawford F , Kappler JW , Marrack P . Memory CD4 T cells that express CXCR5 provide accelerated help to B cells. J Immunol 2011; 186:2889–96. 21270407 10.4049/jimmunol.1002955 PMC3069687

[67] Morita R , Schmitt N , Bentebibel SE , Ranganathan R , Bourdery L , Zurawski G , *et al* Human blood CXCR5(+)CD4(+) T cells are counterparts of T follicular cells and contain specific subsets that differentially support antibody secretion. Immunity 2011; 34:108–21. 21215658 10.1016/j.immuni.2010.12.012 PMC3046815

[68] Schaerli P , Willimann K , Lang AB , Lipp M , Loetscher P , Moser B . CXC chemokine receptor 5 expression defines follicular homing T cells with B cell helper function. J Exp Med 2000; 192:1553–62. 11104798 10.1084/jem.192.11.1553 PMC2193097

[69] Weber JP , Fuhrmann F , Hutloff A . T‐follicular helper cells survive as long‐term memory cells. Eur J Immunol 2012; 42:1981–8. 22730020 10.1002/eji.201242540

[70] Alexander J , Bilsel P , del Guercio MF , Stewart S , Marinkovic‐Petrovic A , Southwood S , *et al* Universal influenza DNA vaccine encoding conserved CD4+ T cell epitopes protects against lethal viral challenge in HLA‐DR transgenic mice. Vaccine 2010; 28:664–72. 19895924 10.1016/j.vaccine.2009.10.103 PMC3364000

[71] McMurry JA , Johansson BE , De Groot AS . A call to cellular & humoral arms: enlisting cognate T cell help to develop broad‐spectrum vaccines against influenza A. Hum Vaccin 2008; 4:148–57. 18382131 10.4161/hv.4.2.5169

[72] Ise W , Inoue T , McLachlan JB , Kometani K , Kubo M , Okada T , *et al* Memory B cells contribute to rapid Bcl6 expression by memory follicular helper T cells. Proc Natl Acad Sci USA 2014; 111:11 792–7. 10.1073/pnas.1404671111 PMC4136626 25071203

[73] Dogan I , Bertocci B , Vilmont V , Delbos F , Megret J , Storck S , *et al* Multiple layers of B cell memory with different effector functions. Nat Immunol 2009; 10:1292–9. 19855380 10.1038/ni.1814

[74] Onodera T , Takahashi Y , Yokoi Y , Ato M , Kodama Y , Hachimura S , *et al* Memory B cells in the lung participate in protective humoral immune responses to pulmonary influenza virus reinfection. Proc Natl Acad Sci USA 2012; 109:2485–90. 22308386 10.1073/pnas.1115369109 PMC3289300

[75] Strutt TM , McKinstry KK , Kuang Y , Bradley LM , Swain SL . Memory CD4+ T‐cell‐mediated protection depends on secondary effectors that are distinct from and superior to primary effectors. Proc Natl Acad Sci USA 2012; 109:E2551–60. 22927425 10.1073/pnas.1205894109 PMC3458385

[76] Bambery B , Selgelid M , Weijer C , Savulescu J , Pollard AJ . Ethical criteria for human challenge studies in infectious diseases. Public Health Ethics 2016; 9:92–103. 29731811 10.1093/phe/phv026 PMC5926904

[77] Ishizuka AS , Lyke KE , DeZure A , Berry AA , Richie TL , Mendoza FH , *et al* Protection against malaria at 1 year and immune correlates following PfSPZ vaccination. Nat Med 2016; 22:614–23. 27158907 10.1038/nm.4110 PMC11294733

[78] Seder RA , Chang LJ , Enama ME , Zephir KL , Sarwar UN , Gordon IJ , *et al* Protection against malaria by intravenous immunization with a nonreplicating sporozoite vaccine. Science 2013; 341:1359–65. 23929949 10.1126/science.1241800

[79] Epstein JE , Tewari K , Lyke KE , Sim BK , Billingsley PF , Laurens MB , *et al* Live attenuated malaria vaccine designed to protect through hepatic CD8(+) T cell immunity. Science 2011; 334:475–80. 21903775 10.1126/science.1211548

[80] Sissoko MS , Healy SA , Katile A , Omaswa F , Zaidi I , Gabriel EE , *et al* Safety and efficacy of PfSPZ Vaccine against Plasmodium falciparum via direct venous inoculation in healthy malaria‐exposed adults in Mali: a randomised, double‐blind phase 1 trial. Lancet Infect Dis 2017; 17:498–509. 28216244 10.1016/S1473-3099(17)30104-4 PMC6803168

[81] Olotu A , Lusingu J , Leach A , Lievens M , Vekemans J , Msham S , *et al* Efficacy of RTS, S/AS01E malaria vaccine and exploratory analysis on anti‐circumsporozoite antibody titres and protection in children aged 5–17 months in Kenya and Tanzania: a randomised controlled trial. Lancet Infect Dis 2011; 11:102–9. 21237715 10.1016/S1473-3099(10)70262-0 PMC3341451

[82] Reed SG , Orr MT , Fox CB . Key roles of adjuvants in modern vaccines. Nat Med 2013; 19:1597–608. 24309663 10.1038/nm.3409

[83] Olotu A , Moris P , Mwacharo J , Vekemans J , Kimani D , Janssens M , *et al* Circumsporozoite‐specific T cell responses in children vaccinated with RTS, S/AS01E and protection against P falciparum clinical malaria. PLoS ONE 2011; 6:e25786. 21998698 10.1371/journal.pone.0025786 PMC3188575

[84] Rts SCTP , Agnandji ST , Lell B , Soulanoudjingar SS , Fernandes JF , Abossolo BP , *et al* First results of phase 3 trial of RTS, S/AS01 malaria vaccine in African children. N Engl J Med 2011; 365:1863–75. 22007715 10.1056/NEJMoa1102287

[85] De Gregorio E , Rappuoli R . From empiricism to rational design: a personal perspective of the evolution of vaccine development. Nat Rev Immunol 2014; 14:505–14. 24925139 10.1038/nri3694 PMC7096907

[86] Lewinsohn DA , Lewinsohn DM , Scriba TJ . Polyfunctional CD4(+) T Cells as targets for tuberculosis vaccination. Front Immunol 2017; 8:1262. 29051764 10.3389/fimmu.2017.01262 PMC5633696

[87] Gengenbacher M , Nieuwenhuizen NE , Kaufmann S . BCG ‐ old workhorse, new skills. Curr Opin Immunol 2017; 47:8–16. 28719821 10.1016/j.coi.2017.06.007

[88] Sharpe S , White A , Sarfas C , Sibley L , Gleeson F , McIntyre A , *et al* Alternative BCG delivery strategies improve protection against Mycobacterium tuberculosis in non‐human primates: protection associated with mycobacterial antigen‐specific CD4 effector memory T‐cell populations. Tuberculosis 2016; 101:174–90. 27865390 10.1016/j.tube.2016.09.004 PMC5120991

[89] Rerks‐Ngarm S , Pitisuttithum P , Nitayaphan S , Kaewkungwal J , Chiu J , Paris R , *et al* Vaccination with ALVAC and AIDSVAX to prevent HIV‐1 infection in Thailand. N Engl J Med 2009; 361:2209–20. 19843557 10.1056/NEJMoa0908492

[90] Chung AW , Ghebremichael M , Robinson H , Brown E , Choi I , Lane S , *et al* Polyfunctional Fc‐effector profiles mediated by IgG subclass selection distinguish RV144 and VAX003 vaccines. Sci Transl Med 2014; 6:228ra38. 10.1126/scitranslmed.3007736 24648341

[91] Haynes BF , Gilbert PB , McElrath MJ , Zolla‐Pazner S , Tomaras GD , Alam SM , *et al* Immune‐correlates analysis of an HIV‐1 vaccine efficacy trial. N Engl J Med 2012; 366:1275–86. 22475592 10.1056/NEJMoa1113425 PMC3371689

[92] Yates NL , Liao HX , Fong Y , deCamp A , Vandergrift NA , Williams WT , *et al* Vaccine‐induced Env V1‐V2 IgG3 correlates with lower HIV‐1 infection risk and declines soon after vaccination. Sci Transl Med 2014; 6:228ra39. 10.1126/scitranslmed.3007730 PMC4116665 24648342

[93] Borrow P , Moody MA . Immunologic characteristics of HIV‐infected individuals who make broadly neutralizing antibodies. Immunol Rev 2017; 275:62–78. 28133804 10.1111/imr.12504 PMC5299500

[94] Beura LK , Hamilton SE , Bi K , Schenkel JM , Odumade OA , Casey KA , *et al* Normalizing the environment recapitulates adult human immune traits in laboratory mice. Nature 2016; 532:512–6. 27096360 10.1038/nature17655 PMC4871315

[95] Rosshart SP , Vassallo BG , Angeletti D , Hutchinson DS , Morgan AP , Takeda K , *et al* Wild mouse gut microbiota promotes host fitness and improves disease resistance. Cell 2017; 171:1015–28 e13. 29056339 10.1016/j.cell.2017.09.016 PMC6887100

[96] Grassly NC , Fraser C , Wenger J , Deshpande JM , Sutter RW , Heymann DL , *et al* New strategies for the elimination of polio from India. Science 2006; 314:1150–3. 17110580 10.1126/science.1130388

[97] Levine MM , Kaper JB , Herrington D , Ketley J , Losonsky G , Tacket CO , *et al* Safety, immunogenicity, and efficacy of recombinant live oral cholera vaccines, CVD 103 and CVD 103‐HgR. Lancet 1988; 2:467–70. 2900401 10.1016/s0140-6736(88)90120-1

[98] Patel M , Shane AL , Parashar UD , Jiang B , Gentsch JR , Glass RI . Oral rotavirus vaccines: how well will they work where they are needed most? J Infect Dis 2009; 200(Suppl 1):S39–48. 19817613 10.1086/605035 PMC3673012

